# The Role of Social Capital in Successful Adherence to Antiretroviral Therapy in Africa

**DOI:** 10.1371/journal.pmed.1000018

**Published:** 2009-01-27

**Authors:** Agnes Binagwaho, Niloo Ratnayake

## Abstract

Agnes Binagwaho and Niloo Ratnayake discuss the implications of a new ethnographic study that explores the reasons for the high rates of adherence to antiretroviral medicines in Africa.

In the late 1990s and early 21st century, some public health officials in the Western world believed that Africans would never be compliant with antiretroviral therapy (ART) because the continent's uneducated, illiterate population was driven by day-to-day concerns without much thought for the long-term future. On top of this, many claimed that ART was a luxury for Africans and that the complex disease would be too difficult for African doctors to manage in the middle of nowhere with no water or electricity. Some went so far as to insist that giving ART to a likely noncompliant population would create drug resistance and were willing to sacrifice Africa for the good of global public health [1].

However, recent research has shown that levels of adherence to ART in sub-Saharan Africa are in fact *higher* than those in North America [2]. Why are Africans with HIV more adherent to ART than their counterparts in North America despite being less educated about HIV and having more obstacles to overcome? A new study by Norma Ware and colleagues published in *PLoS Medicine* sets out to answer this complicated question [3]. This study investigates the surprising finding that Africans would want to take drugs that would give them life and keep them healthy. Anthropologically, this first question leads to another complex question: Why is it that when things are successful in Africa the rest of the world looks for a reason, but when things fail, there are few who question the failure?

## The New Study

Ware and colleagues asked patients, treatment partners (those who assisted patients in their efforts to take antiretroviral medications), and health care providers what the driving factors were behind adherence in three sites: (1) The Immune Suppression Syndrome clinic at Mbarara University of Science and Technology in Mbarara, Uganda; (2) the ART clinic of Amana District Hospital in Dar es Salaam, Tanzania; and (3) the HIV/AIDS clinic at Jos University Teaching Hospital in Jos, Nigeria. Patient interviews focused on experiences with taking ART, clinic visits, and help from treatment partners. Treatment partner interviews targeted the types of help that they give, feelings about being a treatment partner, and opinions on their impact. Health care workers, such as clinicians, nurses, and others, were asked to describe typical clinic visits, ways adherence is discussed at these visits, and views of patient obstacles towards adherence. The researchers also conducted observations of clinic visits, with a focus on observing routine follow-up visits of patients taking ART, counseling sessions, health education sessions, and the dispensing of antiretroviral medications. In all, 158 patients, 45 treatment partners, and 49 health care workers were interviewed. There were 414 interviews and 136 observation sessions conducted across the three sites.

**Linked Research Article**

This Perspective discusses the following new study published in *PLoS Medicine*:

Ware NC, Idoko J, Kaaya S, Biraro IA, Wyatt MA, et al. (2009) Explaining adherence success in sub-Saharan Africa: An ethnographic study. PLoS Med 6(1): e1000011. doi:10.1371/journal.pmed.1000011

Using ethnographic data from Nigeria, Tanzania, and Uganda, Norma Ware and colleagues examine why levels of adherence to HIV/AIDS drugs are so much higher in sub-Saharan Africa than in North America.

The study shows that Africans overcome economic obstacles to get ART by begging and borrowing money from friends, families, and even their health care providers. They may choose to use money for transportation for their clinic appointments over food for them or their family, over school fees for their children, and over treatment for their sick child. Patients without money would take their medications without food despite the increased risk of side effects and would walk to health clinics despite long distances. Health care providers also made sacrifices by keeping their offices open longer to accommodate patients who arrived late and gave food, money, and even loans to needy patients.

The study also shows the significant impact of social responsibility upon adherence. Families and friends often help finance health needs, but this assistance becomes more difficult to justify if it is believed the patient is near death or incurable. Treatment partners insisted on adherence to ART by those they cared for because it made the treatment partner's burden lighter. The treatment partners' help created an obligation to patients to be adherent. Some health care providers threatened to discontinue giving treatment to nonadherent clients, further emphasizing the social responsibility patients had to remain adherent.

## Public Health Implications

Ware and colleagues' multi-country, multi-setting study used a methodology of interpersonal interaction that allowed people to talk simply to the researchers, without complicated study designs getting in the way. Instead of coming with experts and boxes to check, the researchers captured the viewpoint of the patient, treatment partner, and grassroots-level health care worker. The findings show the importance of social capital (the connections between people) and reveal that social responsibility in Africa pushes people to be “good” (adherent) patients. Social capital has been used in other countries such as Rwanda, where those who want treatment must come to the clinic with a relative or members of their association.

Those who have truly been working in Africa should not have learned anything new. Social capital can be easily seen in day-to-day health care work. This new study will, however, hopefully change the way the rest of the world views Africa. The study proves that human rights activists were correct in believing that giving ART in underdeveloped settings does not put the world in danger. When future myths about Africa are presented, people can point to this study to show that the uneducated, illiterate, and poor still want to survive, perhaps even more than those in Europe or the United States. Although this study does not change policies and will not affect future clinical decisions, it can be a useful tool to create support for access to treatment in Africa.

Social coercion in Africa is high because people are more responsible for each other. Yes, Africans want to live, but more than that, they want to keep their relationships alive. Patients living with HIV in North America often have negative experiences and complicated backgrounds, such as traumatic events and mental illness, with little social support, and they face a great deal of stigma [4]. Social capital is less strong in the United States than in Africa as people in the US tend to be more individualistic and therefore less focused upon and connected to the group as a whole. There is less concern about others and less of a feeling that others are concerned about you. Ware and colleagues state that: “In North America, adherence to ART for HIV/AIDS has been interpreted as the product of information, motivation, and behavioral skills operating at the individual level.” In other words, the driving factor for Americans to take their drugs is not social responsibility, but intellectualization of what to do to remain alive. Patients take the drugs they are given for themselves and not for others. Therefore, when a patient becomes depressed, adherence often declines. Research has shown that depressed people living with HIV progress to AIDS faster than those who are not depressed, due in part to nonadherence [5]. In Africa, on the other hand, taking prescribed ART is a *community* effort. Even when patients no longer care for themselves, they continue to take the medication for the community around them.

## Next Steps

Having clearly established the importance of social capital in promoting adherence to ART, future studies should focus both on its protective effects outside of the scope of HIV/AIDS and on how to maintain social capital while improving economic development, which can bring a more individualistic way of life. A developed economy provides an environment that allows the growth of individualism, while the poor tend to depend on their community for survival. Social capital can be a useful tool in promoting adherence to medications in patients with chronic diseases, who are at higher risk of depression than the general population [6]. As countries in Africa become more economically developed, it will be increasingly more important for them to actively find tools to maintain their social capital.

## Abbreviations

ART: antiretroviral therapy
